# Anticodon Modifications in the tRNA Set of LUCA and the Fundamental Regularity in the Standard Genetic Code

**DOI:** 10.1371/journal.pone.0158342

**Published:** 2016-07-25

**Authors:** Peter T. S. van der Gulik, Wouter D. Hoff

**Affiliations:** 1 Centrum Wiskunde & Informatica, P.O. Box 94079, 1090 GB Amsterdam, The Netherlands; 2 Department of Microbiology and Molecular Genetics, Oklahoma State University, Stillwater, Oklahoma, 74078, United States of America; 3 Department of Chemistry, Oklahoma State University, Stillwater, Oklahoma, 74078, United States of America; Niels Bohr Institute, DENMARK

## Abstract

Based on (i) an analysis of the regularities in the standard genetic code and (ii) comparative genomics of the anticodon modification machinery in the three branches of life, we derive the tRNA set and its anticodon modifications as it was present in LUCA. Previously we proposed that an early ancestor of LUCA contained a set of 23 tRNAs with unmodified anticodons that was capable of translating all 20 amino acids while reading 55 of the 61 sense codons of the standard genetic code (SGC). Here we use biochemical and genomic evidence to derive that LUCA contained a set of 44 or 45 tRNAs containing 2 or 3 modifications while reading 59 or 60 of the 61 sense codons. Subsequent tRNA modifications occurred independently in the Bacteria and Eucarya, while the Archaea have remained quite close to the tRNA set as it was present in LUCA.

## Introduction

### Towards analyzing the middle stage of the evolution of the SGC

The evolutionary origin of the standard genetic code (SGC) is widely viewed as a central open problem in the evolution of life [1–4]. Key questions in the field focus on early steps in the evolution of the SGC, such as: what is the origin of the first tRNA and what is the amino acid that it encoded; how did this first tRNA give rise to a set of 20 encoded amino acids? Here we consider events in a later stage of the evolution of the code involving anticodon modifications that affect the readout properties of tRNAs. With the availability of complete genomes of hundreds of organisms from all three domains, the possibility emerges for a meaningful investigation of the tRNA set of the last universal common ancestor (LUCA). Here we will focus on reconstructing the anticodon modifications which were used in the tRNA set of LUCA.

While the genetic code is at the core of all known cellular life, its evolutionary origin remains only very partially understood. We propose to distinguish three stages in the evolution of the genetic code. During the first stage, the genetic code emerged and evolved from a system with few amino acids to a system with the current twenty amino acids. This stage involved a small number of tRNAs and no anticodon modifications, and as a result not all codons were read efficiently (see below). During stage 2 (the “middle stage”), the readout properties of the tRNA sets were improved through evolutionary development of modifications of the bases in the anticodon. In addition, release factor proteins evolved to increase the efficiency of translational termination. With the help of anticodon modifications all 61 sense codons could be recognized quickly and unambiguously by the tRNA set. These events resulted in the evolution of the modern SGC. Currently it is not clear if this second stage was already completed in LUCA. During stage 3, small variations in the SGC evolved in a limited number of present-day lineages. All of these minor code variations are present in relatively small, taxonomically coherent groups of current organisms and their origin can be traced back to a small modification in the genetic code in a relatively recent common ancestor that carried the SGC [5–7]. These genetic code variants therefore arose post-LUCA during the last ~3 billion years. The evolution of such a new code variant occurs during a relatively short period in which the “frozen accident” [8] of the SGC briefly thaws between long eras of codon assignment stasis. The proposed stages 1 and 2 of genetic code evolution (during which the SGC emerged and froze) occurred almost completely (see below) pre-LUCA during the first ~1.5 billion years of earth’s history.

### Biochemical understanding of the fundamental regularity in the SGC

A large body of literature exists on both stages 1 and 3. Stage 1 is the most challenging to address. Stage 3 has been well documented and is well understood (see e.g. [9] and references therein). However, little attention has been paid to stage 2. In [10], we drew attention to the fact that a relatively small tRNA set with unmodified anticodons is able to unambiguously read more than 80% of the codons of the genetic code. As discussed below, evidence has accumulated [11–14] that an important fundamental regularity exists in the SGC, which provides key constraints on its evolutionary origin. Here we further explore the implications of this regularity in the SGC for the evolutionary pathway that resulted in its development.

**Fig 1:**
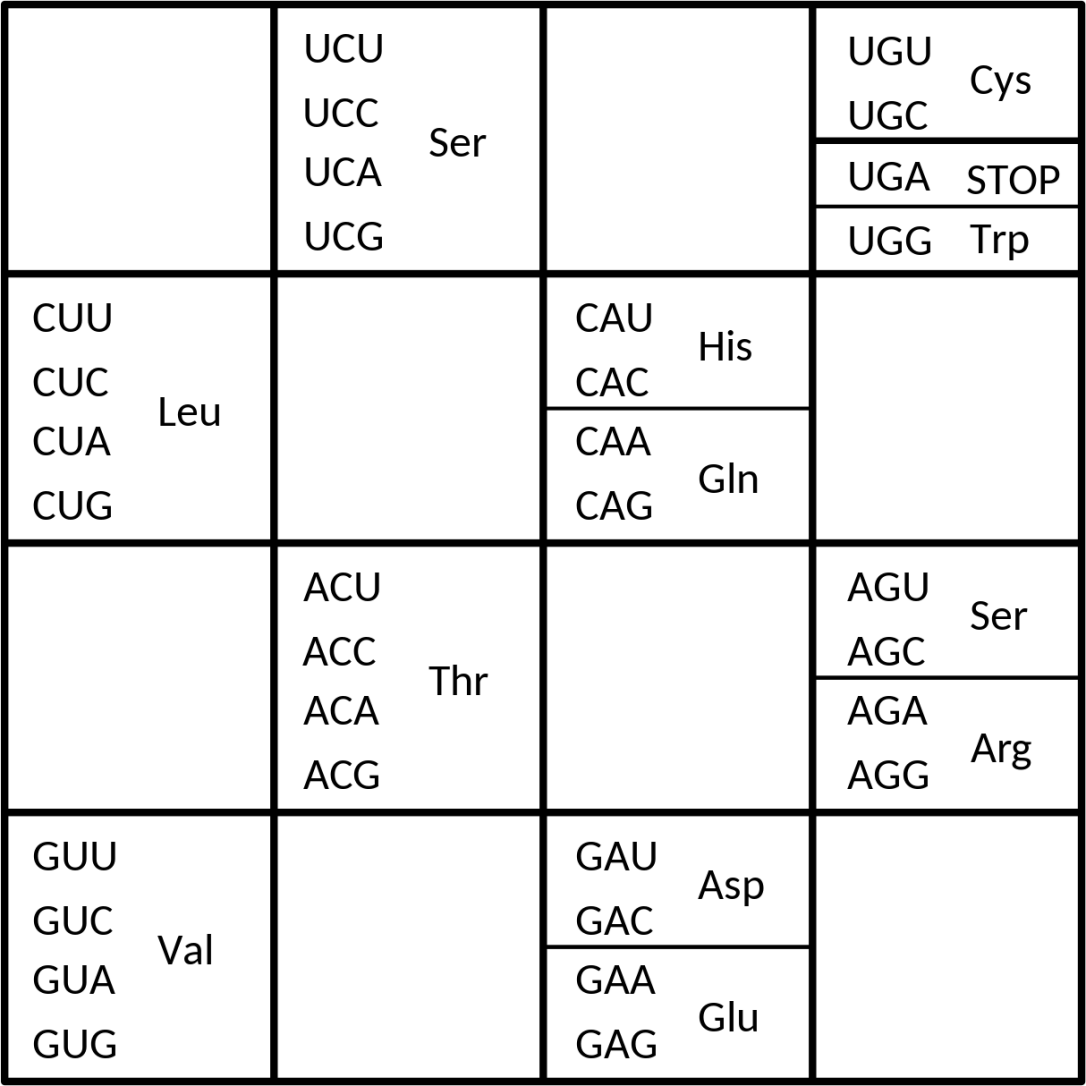
The chess board pattern in the genetic code table. When all SSN and WWN codon boxes are left out, a chess board pattern emerges (see text). In this representation it can immediately be seen that mixed SW/WS codon boxes with a middle-Y (U or C) are fourfold degenerate codon boxes, while mixed SW/WS codon boxes with a middle-R (A or G) are split codon boxes.

We focus on the regularity that the 16 codon boxes (defined as the set of four triplets sharing the first two nucleotides) are divided exactly in two groups of 8 codon boxes each: the 8 fourfold degenerate codon boxes, and the 8 split codon boxes. Furthermore, this neat division in two groups of 32 codons is not a random division: to the contrary, it is extremely regular. The 4 SSN codon boxes (where S stands for G or C) all belong to the fourfold degenerate group of codon boxes; the 4 WWN codon boxes (where W stands for A or U) all belong to the group of split codon boxes. The extremely regular division also extends to the remaining 8 codon boxes. These are characterized by a mix of S and W nucleotides in the first two codon positions and form a “chess board pattern” in the genetic code table (see Fig 1). The four NYN codon boxes (Y denotes a pyrimidine: C or U) from this group (UCN, CUN, ACN, and GUN) all belong to the group of fourfold degenerate codon boxes. The four NRN codon boxes (where R denotes a purine: A or G) from this group (UGN, CAN, AGN, and GAN) all belong to the group of split codon boxes. The “chess board” is therefore precisely divided into a left half and a right half: the UCN, CUN, ACN, and GUN codon boxes in the left half are fourfold degenerate codon boxes, and the UGN, CAN, AGN, and GAN codon boxes in the right half are split codon boxes. The role of S and W nucleotides in this exact division points to the role of codon-anticodon pairing strength in this regularity.

This regular pattern of exactly splitting the 64 codons into 32 codons belonging to 8 fourfold degenerate codon boxes and 32 codons belonging to 8 split codon boxes was pointed out in 1966 [15]. However, apart from a number of notable exceptions (e.g. [14,16,17]), it has since mostly been ignored. Here we return to this regularity and explore its implications for the evolution of the SGC.

In an important contribution, Lehmann and Libchaber [11] explained the *molecular* raison d’etre of this presence of two types of codon boxes, distributed exactly evenly over the code table: a stabilizing hydrogen bond from U_33_ towards the middle purine of the anticodon (note that a middle pyrimidine in the codon interacts with a middle purine in the anticodon) is responsible for the ability of U-starting anticodons (with unmodified U) in the UCN, CUN, ACN, and GUN codon boxes to read all 4 codons with approximately equal efficiency [11]. In split codon boxes with mixed S and W nucleotides in the first two positions, such a fourfold superwobble [18] is not possible. In these codon boxes, U-starting anticodons (with unmodified U) efficiently pair with the R-ending codons, but not with the Y-ending codons. This effect does not result in a total absence of pairing: suppression (see below) can happen when no able competitor for pairing is present (see [19,20]).

The superwobble (which does not involve suppression) was biochemically demonstrated for tRNA^Gly^_UCC_ [12] and was shown to be dependent on the phenomenon of bridging water molecules between the two bases involved in molecular dynamics simulation [13]. These bridging water molecules provide an appealing explanation of the failure of Crick’s classic argument that Y-Y pairs would be too short (see the legend of Fig 6 in [21]:”The wobble code suggested uses the four positions to the right of the diagram, but not the three close positions”). From a genomics perspective, the superwobble was demonstrated to be present in many fourfold degenerate codon boxes in many bacterial species [14]. The *evolutionary* raison d’etre of the exact division (i.e. bifurcation) in fourfold degenerate codon boxes and split codon boxes has not been addressed and is examined here.

## Results and Discussion

### Evolutionary origin of the fourfold degenerate/split codon box regularity in the SGC based on wobble behavior of tRNA sets with unmodified anticodons

Here we examine the implications of the wobble behavior of tRNAs with unmodified anticodons for the evolutionary origin of the regularity in number and amino acid assignment of the two types of codon boxes in the SGC. In developing molecular scenarios for this stage of the evolution of the genetic code we use three general considerations. First, it appears likely that during the earlier stages of the evolution of the SGC the accuracy of genome repair and replication systems was substantially lower than it is in most present-day cells. As a result, because of the looming of Eigen and Schuster’s error catastrophe [22], strong selective pressure existed to achieve all cellular processes, including translation, with as few components as possible.

The importance of both the speed and accuracy of translation as selective pressures that drive codon bias in present-day organisms has been established [14]. Here we argue that during the earlier stages of the evolution of the SGC a third selective pressure played a major role: to perform translation with components using the smallest possible genome size. This argument hinges on the notion that during these stages of evolution of the genetic code the effective genome size of these early systems was considerably smaller than in present-day organisms. The importance of this consideration for the work reported here is that it favors scenarios in which no machinery for base modifications is involved, resulting in the occurrence of tRNAs with unmodified anticodons.

As a second principle in guiding the development of scenarios for the early evolution of the genetic code, we invoke the stabilizing effect resulting from the occurrence of a sufficiently large number of codons in a genome. When codons are present in sufficiently large numbers, protein residues occur where the presence of a certain amino acid side chain is essential. Even if only one such position is vital, the system cannot survive without the ability to translate this position in the correct way. As a result, the SGC is quite stable. This concept has become well known as part of the frozen accident theory [8], but actually is older [23] (also see [4]). The principle that a feature that is in general use cannot be lost without severe consequences was elaborated more recently [24]. The stabilizing effect of this principle on the genetic code is referred to as the proteomic constraint on the genetic code. This proteomic constraint is proportional to the size of the proteome, measured as P, the number of codons in a genome [24]. If P is small (e.g. smaller than 100,000, which is the case for the set of 13 protein-encoding genes in a mammalian mitochondrial genome), then changes in the genetic code can occur relatively easily (see also [9] and references therein).

Third, in the evolutionary scenario reported here we consider the phenomenon that to a certain extent anticodons are able to read (albeit with reduced efficiency) codons outside their canonical group of codons. This effect has been experimentally observed in cases in which a specific tRNA is absent but the codons that are canonically read by the missing tRNA are being read by an alternative anticodon. This process is referred to as the suppression of a potentially highly detrimental situation in which a codon is formally unassigned but in fact is translated by a different tRNA through non-canonical codon-anticodon pairing. Please note that the use of the term suppression is potentially confusing (see [25]). The term suppression by tRNAs was originally used to describe suppressor mutations in which a lethal mutation to an in-frame stop codon was suppressed by a mutation in a tRNA that allowed the in-frame stop codon to be read as a sense codon. Söll and co-workers [25] refer to “introducing new amino acid assignments of one or more codons without removing the original function (e.g. UAG decoded as both a stop codon and an amino acid [26])” as codon suppression. In this study, we use the term suppression for those cases in which a tRNA reads (with reduced efficiency) a codon outside its normal group of recognized codons.

Based on the evolutionary pressure to perform translation with the smallest effective tRNA set, we propose that early in the evolution of the genetic code all 4 codons in a fourfold degenerate codon box were translated by the U-starting anticodon as the single anticodon. Strong biochemical evidence for this proposal has been reported [12]. For example, in the scenario proposed here the UCN codon box was translated by a single tRNA with anticodon UGA. In the case that the UGA anticodon mutated to one of the three other anticodons (GGA, AGA, CGA) working in the same codon box, a suboptimal situation had come into existence. If, in the example of the UCN codon box, the anticodon changed from UGA to GGA, the UCR codons were not read efficiently because a tRNA with the GGA anticodon was less adapt at reading UCR codons compared with UGA. While G-starting anticodons in many cases can suppress A-ending codons (see [27]), translation is often impaired. For G-ending codons, suppression by G-starting anticodons is problematic. These biochemical results on present-day organisms (see e.g. [27,28]) indicate that the UGA anticodon in fact was present at a much higher frequency than its GGA, AGA or CGA variants during early stages of the evolution of the genetic code. This frequency distribution resulted from the balance between point mutations leading to the introduction of these anticodons and negative selection leading to their removal.

The relevance of the proteomic constraint for the evolution of the genetic code as considered here is that the presence of vital UCR codons in a genome will cause the mutation of the UGA anticodon to GGA to strongly reduce fitness. Therefore, in fourfold degenerate codon boxes, where one single tRNA without anticodon modification is able to efficiently read all 4 codons, during the early evolution of the genetic code the proteomic constraint on the genetic code maintains the first position of the anticodon as U.

In contrast to the situation described above for fourfold degenerate codon boxes, in the case of split codon boxes (such as UUN), biochemical considerations indicate that the U-starting anticodon was not the preferred anticodon. The key factor is that when the superwobble is not possible, selection during the early evolution of the genetic code will favor the presence of two distinct tRNA genes, one with a G-starting anticodon which will efficiently read the Y-ending codons, and one with a C-starting anticodon which will efficiently read the G-ending codon. Diversification of amino acid assignment can then follow. In this scenario the presence of a tRNA with U-starting anticodon will be harmful: although not able to efficiently read the Y-ending codons, it will sufficiently suppress them to create damaging ambiguity in their translation. As the proteome evolves and becomes more sophisticated, ambiguity becomes an increasingly important problem.

Two distinct biochemical approaches can be envisioned. First, the evolution of a machinery for anticodon modification could resolve the translational ambiguity of U-starting anticodons for split codon boxes. Second, the molecular solution to this translational ambiguity is to avoid the U-starting anticodon and to restrict the anticodons to G-starting and C-starting variants (for the split codon boxes). In this second approach, the negative selection on the presence of U-starting anticodons in split codon boxes also occurs for the presence of A-ending codons (in split codon boxes) [10]. With only G-starting and C-starting anticodons present, the A-ending codons cannot be read efficiently, and will become rare in the genome. This is a direct effect of the positive selection on reduction of ambiguity: the ability to efficiently read A-ending codons was less important than the power to use unambiguous codons. Thus, developing unambiguous coding came with the cost of the inefficient translation of A-ending codons (in the split codon boxes), and therefore negative selection on the presence of these codons in protein-encoding sequences.

A key issue for the scenario developed here is to evaluate which of the two above approaches is more likely to have occurred. A concern regarding the second scenario is that A-ending codons in the split codon boxes remain formally unassigned. This issue is considered below. An appealing aspect of the second approach is that it negates the need for additional components in the translational system that would be needed to achieve suitable anticodon modification, and that it builds on the striking property of entirely unmodified anticodons to translate all 20 amino acids while reading 55 of the 61 sense codons. We prefer the second biochemical approach because evolution of a machinery for anticodon modification is, in our view, a phenomenon which belongs to a later stage of evolution in which a larger genome and a more sophisticated enzyme collection are present.

A recurring line of thought in published work on the evolution of the SGC is that the presence of formally unassigned codons is extremely damaging to the organism [8, 29–32]. In this argument these codons are essentially untranslatable, and when unavoidable random mutations result in the introduction of these codons, they function as nonsense mutations. Experimental evidence in present-day organisms has demonstrated that the presence of formally unassigned codons even in essential genes can leave cells viable through the process of suppression. We proposed that such suppression would also reduce the damaging nature of the above nonsense mutations. This consideration argues in favor of scenarios for the evolution of the SGC in which the occurrence of formally unassigned codons is allowed. An alternative scenario in which not C-starting anticodons but U-starting anticodons occur offers the advantage that the A-ending codons are formally assigned. However, this approach comes at the cost of the ambiguous translation of Y-ending codons through suppression of unmodified U-starting anticodons. In our assessment, the fitness cost to an organism of this chronic ambiguous translation is higher than the cost of infrequent nonsense mutations to formally unassigned (but suppressed) A-ending codons.

A possible concern regarding the scenario proposed here is that in contemporary tRNA sets currently no organisms are known that use C-starting anticodons while not having U-starting anticodons (and therefore not using A-ending codons with substantial frequencies). However, the evolutionary events considered here are of extremely ancient character (pre-LUCA). As a result, it appears entirely plausible that the rarity of A-ending codons in split codon boxes of the earlier tRNA set proposed can have been erased in present-day organisms. Please note that AGA codons in fact are rare in bacteria (see e.g. [33]. Another aspect of the unassigned A-ending codons in split codon boxes is that it is possible that they did not go down in numbers, but that they had never been present in any large numbers in the earlier phases of genetic code evolution. The number of codons in frequent use can have been steadily growing during the evolutionary development of the code. The A-ending codons in split codon boxes might simply not have been assigned yet when C-starting anticodons started to assign G-ending codons in split codon boxes. The viewpoint that “as soon as a small set of amino acids started to be encoded by tRNAs, rapid tRNA gene duplication and mutation of the anticodon resulted in a situation in which all codons were assigned to this initial set of amino acids” is not unchallenged (see [10]). A much more gradual growth of the number of assigned codons, without much of the damaging reassignment, is a *bona fide* alternative for the view of rapid assignment of all codons in the code table (which mainly goes back to Crick [8] and Jukes [34]).

Interesting in connection to our proposal of primordial rarity of A-ending codons in split codon boxes is the work of Trifonov (e.g. [35]) about triplet expansion diseases. Simple sequence repeats, of which the triplet repeats are an example, are known in both vertebrates and bacteria. “It is generally assumed that during transcription, transient pausing of the RNA polymerase complex promotes backward slippage and leads to resynthesis of the same RNA sequence” [36]. Trifonov proposes that this phenomenon connected to RNA production is much more general (than only being present in bacterial immune-escape and in vertebrate expansion disease) and was particularly abundantly present during the very first stages of genetic code development. In his view Gly and Ala would be the sole amino acids in use in very early life, GCC and GGC codons would be the codons for these amino acids, and triplet expansion (GCC being one of the codons known for triplet expansion, and GGC being its complementary codon) would lead to longer RNAs. A consequence of such a start of genetic code development is a high abundance of G-starting codons and a low abundance of A-ending codons as an original characteristic of protein coding sequences. Trifonov is not the first nor the only one to suggest primordial GNN richness of protein coding sequences, cf. [37,38] and references in [38].

Taken together, the arguments that we present above argue that the cost of leaving codons formally unassigned is likely to be much smaller than is generally assumed. And, secondly, that (as we propose) the costs of devoting precious genome space to additional components needed for anticodon modifications at early stages of the evolution of the genetic code is much higher than is often assumed. This assessment of the costs and benefits of the two scenarios is distinct from most published work in the area of the evolution of the genetic code. These considerations lead us to conclude that the second scenario, in which the U-starting anticodons are avoided in split codon boxes, should be considered the preferable one.

The above considerations indicate that subtle differences in the stability of codon-anticodon complexes between those of the fourfold degenerate codon boxes and those of the split codon boxes and the inability of U-starting anticodons in split codon boxes to perform an efficient fourfold degenerate wobble had crucial consequences for developing evolutionary stable tRNA sets during the early evolution of the genetic code. This view of the evolutionary development of tRNA sets and the susceptibility of tRNA sets by novel tRNAs created through point mutations in the anticodon is an application of the concept of an evolutionary stable strategy [39–42] to tRNA sets during the evolution of the SGC. A consequence of this effect is that all 4 codons in the UCN codon box were translated by a single tRNA containing a UGA anticodon. A corresponding line of reasoning applies to the other seven fourfold degenerate codon boxes. In contrast, the UUN codon box was translated by two different tRNAs containing CAA and GAA anticodons. As a result of the inability to perform an efficient fourfold wobble, UAA was not evolutionarily stable as a Leu anticodon while CAA was stable as a Leu anticodon. This argument also applies to the other seven split codon boxes. These considerations provide the first evolutionary explanation for the fundamental structure of the genetic code (8 fourfold degenerate codon boxes and 8 split codon boxes in an extremely regular distribution). Lehmann and Libchaber already pointed out that: “The basic assumption on which the model is built is that wobbling was initially maximized” [11]. That brief statement is in line with the scenario presented here, but does not address why unmodified U-starting anticodons in the split codon boxes were problematic during the early evolution of the code, or what the role of the error catastrophe, the proteomic constraint, and suppression were in this process.

The consequences of the scenario proposed above are that 8 G-starting anticodons (GAA reading UUY, GUA reading UAY, GCA reading UGY, GUG reading CAY, GAU reading AUY, GUU reading AAY, GCU reading AGY, and GUC reading GAY), 8 U-starting anticodons (UGA reading UCN, UAG reading CUN, UGG reading CCN, UCG reading CGN, UGU reading CAN, UAC reading GUN, UGC reading GCN, and UCC reading GGN) and 7 C-starting anticodons (CAA reading UUG, CCA reading UGG, CUG reading CAG, CAU reading AUG, CUU reading AAG, CCU reading AGG, and CUC reading GAG) can achieve a system encoding 20 different amino acids while reading 55 of the 61 sense codons of the SGC [10]. This set of 23 tRNAs in total is able to operate in the absence of any anticodon modifications. Note that according to this viewpoint both Trp and Met have always been coded by one codon (until the advent of recent code variants) and that the third Ile codon (AUA) is a comparatively recent acquisition of Ile (by convergent evolution in archaea and bacteria). The fact that the AUA codon was captured by Ile in *both domains* points to primordial suppression of AUA codons by the anticodon GAU, leading to non-canonical Ile-coding in the primordial genome by AUA at a low frequency. This conclusion is reminiscent of the finding that some modern codon reassignments such as “UGA becomes Trp” and “UAR becomes Gln” are also known to have occurred repeatedly, pointing to the occurrence of suppression before the reassignment event, therefore directing the choice of the reassignment i.e. which reassignment will be positively selected. Evidence in support of such A-ending codon suppression by G-starting anticodons in present-day organisms has been reported [14]. The emergence of a release factor recognizing UGA was the circumstance responsible for the fact that Trp did not grow from one to two codons during subsequent developments of tRNA sets and anticodon modifications. However, we know this reassignment from modern genetic code variants, and in these reassignments first the release factor must disappear. The primordial suppression of AUA codons by anticodon GAU was the circumstance responsible for the fact that Met did not grow from one to two codons. We propose that Gln, Lys, Glu, Leu (coded by UUG), and Arg (coded by AGG) *did* grow from one to two codons, and that the codon recognition characteristics of C-starting anticodons enabled early developing life to liberate itself of ambiguousness in coded oligopeptide synthesis.

The situation of 23 tRNAs described in the paragraph above forms the starting point of stage 2 (introduced above) of the evolution of the SGC. In the highly sophisticated biochemistry of present-day organisms, anticodon modifications provide a range of subtle advantages. However, in the much less sophisticated early genetic code world, tRNA anticodon modifications would be required only to translate the following 6 codons: AUA, AGA, UUA, CAA, AAA, and GAA. Below we use a comparative genomics approach to determine which of the tRNA modifications required for translating these 6 codons were already present in LUCA, and which tRNA modifications evolved post-LUCA. Subsequently, we consider the tRNA set of LUCA.

### Unraveling the final steps in the evolution of the SGC based on diversity in anticodon modification systems

Archaea and Bacteria separately evolved the ability to unambiguously recognize the AUA codon [10]. The use of lysidine at the wobble position by Bacteria has long been known [43]. The use of agmatidine, a chemically different modification, by Archaea was reported much more recently [44]. The two modification enzymes involved are quite distinct [45]. Subsequent experimental biochemical work confirmed the convergent evolution of the translational readout of AUA as an Ile codon [46,47]. Having recognized the manner in which AUA entered the genetic code, we now ask the question: what was the process through which the remaining 5 sense codons (AGA, UUA, CAA, AAA, and GAA) were included in the genetic code? In the case of AGA we found that present data did not allow us to draw a firm conclusion. In bacteria, AGA is a rare codon (see e.g. [33]). In archaea, AGA codons are not rare (see e.g. [48]), but it is not known at this moment which modification of the UCU anticodon in archaea is responsible for preventing suppression of AGY codons. We are forced to leave this as an open question: it is unclear if AGA was already used in LUCA. In the case of UUA, CAA, AAA, and GAA we were able to derive specific conclusions. For each of these codons we will consider if the modification of the anticodon of the tRNA required for their readout developed independently in Archaea and Bacteria, or if the readout of these codons was already optimized in LUCA.

#### UUA

Grosjean and co-workers [27] have drawn attention to the fact that methylation of the ribose part of U_34_ of anticodon UAA is found in all three domains. This chemically simple modification (a single methylation) probably allows the use of A-ending codons (UUA codons in this case) without concomitantly producing unacceptable suppression of Y-ending codons. Later additional modifications fine-tuned translation, and produced a complicated situation, and these modifications are different for different taxonomic groups (see [27]: “The efficacy of U_34_:G_3_ wobbling will strongly depend on the presence of chemical adducts on the *C5* atom of U_34_ […]. Because these enzymatic modifications of U_34_ in naturally occurring tRNAs differ in the three biological kingdoms…”; also see [49]). However, the first step was comparatively simple. Our proposal is that this methylation step allowed the C-starting anticodon CAA to be replaced by the U-starting anticodon UAA, and the UUA codon to become a regular sense codon instead of a rare sense codon.

The fact that the SPOUT methyltransferase TrmL (previously named YibK), which in *E*. *coli* is responsible for this ribose methylation, is one of the smallest SPOUT enzymes known [50], supports the concept that this is an enzyme that was already present in LUCA. Very small enzymes are good candidates to be very old enzymes. This argument is related to the concept of urzymes of Carter and co-workers [51], which have a size of less than 150 amino acid residues. Besides Um (the methylation of the ribose part of U) other modifications of U are known, like mnm^5^U, mcm^5^U, and mcmo^5^U, but these are complex, produced by sets of larger enzymes, and not universal (see e.g. [27], especially Fig 1C), and therefore are, in our opinion, unlikely to have been present in LUCA (see also [27]).

#### CAA, AAA, and GAA

These three codons share the characteristic that their middle nucleotide is A. The fourth A-ending codon with a middle-A (UAA) is a stop codon in the SGC. In present-day organisms the recognition of this A-ending codon as “stop” without the problem of suppression of the Y-ending codons is achieved through a release factor protein. An analysis of the emergence of release factors in LUCA and how these factors evolved in the three domains of life following the approach presented here for the evolution of anticodon modifications appears possible, but is outside the scope of this article. The three codons CAA, AAA, and GAA are discussed as a group because the evolution of a *single* anticodon modification enzyme (which recognizes middle-A anticodons, but does not distinguish CAA, AAA, and GAA) was able to solve the suppression problems associated with the use of these A-ending codons.

Present-day organisms use the 2-thio-modification of U in the first anticodon position (U_34_) to unambiguously read R-ending codons in the middle-A column (see e.g. [52]). Further modification of this residue differs among the three domains (see e.g. [27]), but the use of 2-thio-U is universal. Although the enzymatic route to deliver sulfur differs among different taxonomic groups (see [53] and references therein), the final enzyme thiouridylating the first nucleotide of the UUG, UUU, and UUC anticodons is always an enzyme which first activates the U by adenylation, and then thiolates the residue (see e.g. [52]). In the archaeon *Methanococcus maripaludis*, the 2-thiouridylase has been found to be not orthologous to MnmA, the *Escherichia coli* enzyme, but to be a paralogue, more related to the 2-thiouridylases which modify e.g. C_32_ instead of U_34_ [54]. It is important to keep in mind that sulfur assimilation has undergone an enormous upheaval since the times of LUCA. While current aerobic organisms use sulfate as the sulfur source of sulfur assimilation, in LUCA’s time sulfate was not present due to the (largely) anaerobic circumstances. Therefore, the sulfur relay systems in organisms like *E*. *coli* and *Saccharomyces cerevisiae* likely were not present during LUCA’s time. Cysteine was not the intracellular sulfur source: cytoplasmic Cys levels were very low, and Cys-tRNA^Cys^ was likely produced from phosphoseryl-tRNA^Cys^ [55]. Rauch et al. [56] have argued that homocysteine biosynthesis was used to assimilate sulfur (from sulfide), and Met and Cys derived their sulfur atoms through that pathway. This argument provides a compelling explanation for the pervasive differences in the enzymes in sulfur metabolism in different taxonomic groups [56]. Nevertheless, the use of the 2-thio-modification in the first position of the U-starting anticodons of tRNA^Glu^, tRNA^Lys^ and tRNA^Gln^ is universal.

The universal presence of the 2-thio-modification in U-starting anticodons reading R-ending middle-A sense codons together with the relative chemical simplicity of this modification argues that LUCA already contained 2-thio-U. The relative simplicity of this modification has also been invoked in literature on the role of this modification in the early RNA world (see e.g. [57]). Starting from the view that originally unmodified C-starting anticodons were used to read the G-ending middle-A codons, and taking into consideration that the 2-thio-uridylation is universal, we conclude that in the tRNA set of LUCA the CUG, CUU, and CUC anticodons were already replaced with UUG, UUU, and UUC anticodons that were 2-thio-uridylated at their first nucleotide. The readout of CAR, AAR, and GAR codons was therefore already largely optimized in LUCA.

At this point we warn against a static view in which certain anticodons are always constitutively modified in the same manner. An important example is that the 2-thio modification in *S*. *cerevisiae* is associated with specific patterns of gene expression. Laxman et al. [58] point out that genes highly enriched for the codons AAR, GAR, and CAR are substantially overrepresented in rRNA processing, ribosomal subunit biogenesis and other translation-/growth-specific biological processes. Absence of the 2-thio modification of the anticodons UUU, UUC, and UUG leads to slower translation of mRNAs containing a higher amount of AAR, GAR, and CAR codons, and thus comparatively less translation of the proteins involved in rRNA processing, ribosomal subunit biogenesis and other translation/growth-specific processes. This mechanism results in a controlled reduction in growth rate as the cell faces sulfur scarcity. Please note that in *S*. *cerevisiae* the UUU, UUC, and UUG anticodons are hypermodified, and the ability to read G-ending codons by U-starting anticodons (without concomitant suppression of Y-ending codons) is not solely depending on the 2-thio modification. However, these further modifications are not universal for the three domains of life [27]. The key finding of Laxman and co-workers is that during limitation of Cys and Met, tRNA thiolation is downregulated. Thus, anticodon modification dynamics play a regulatory role in shifts in the proteome via differences in translation speed of mRNAs, due to different codon composition of protein-encoding genes.

In addition to the fundamental importance in evolutionary biochemistry of this new view of tRNA anticodon modification, this dynamic view of cellular tRNA modification status is proving to be of substantial medical importance, including cancer and mitochondrial stress (see e.g. [59–65]). It remains to be investigated if the use of the 2-thio-U modification in gene expression regulation is a more recent phenomenon and specific to Opisthokont (i.e. fungal and animal) cell biology, or if it is a more ancient aspect of life. The use of the elements S, O, and N in signaling (see e.g. [66]), and the use of tRNA anticodon modifications in regulation (see e.g. [67]) are exciting developments in evolutionary biochemistry, and it remains to be determined whether these processes are ancient and universal or comparatively recent and taxon specific.

Having examined the anticodons of the tRNAs reading the codons AUA, AGA, UUA, CAA, AAA, and GAA in LUCA, we next consider the entire tRNA set of LUCA.

### The tRNA set of LUCA and its anticodon modifications

**Fig 2:**
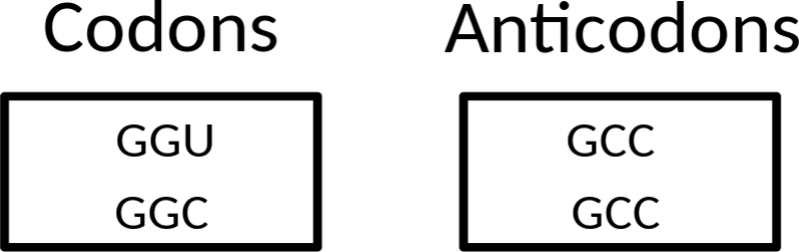
Start of the genetic code with a single tRNA encoding a single amino acid (tentatively selected as Gly). In the left hand panel the codons are indicated which are in efficient and unambiguous use. In the right hand panel the anticodons are indicated which perform the efficient and unambiguous decoding of these codons. The same division between left hand panel and right hand panel is used in Figs 3–6.

In the following paragraphs, we examine the development of the tRNA set of the living cell. The approach followed here considers the biochemical and evolutionary interplay between the tRNA set of an organism and its set of anticodon modification enzymes. This process is reminiscent of the interplay between the evolving tRNA set of an organism and the set of amino acids that it can translate [68,69]. We envision the evolutionary history to have started with a single tRNA (Fig 2) encoding a single amino acid (tentatively selected as Gly), and to have grown by duplications and diversifications of tRNA genes towards the modern tRNA sets of Bacteria, Archaea, and Eucarya. Below we propose three distinct steps during stage 2 of the evolution of the SGC.

As a basis for the scenario of the development of the tRNA set during stage 2 of the evolution of the genetic code, we now consider which key aspects of tRNA sets are universally conserved in all present-day organisms, and which aspects are domain-specific. First, we focus on a feature that is universally conserved in all three domains: **tRNAs with G-starting anticodons in fourfold degenerate codon boxes**. It appears that as genome size was increasing, it became advantageous to have an additional tRNA in the fourfold degenerate codon boxes, taking over the main part of decoding of the Y-ending codons. While in some bacteria with smaller genomes these tRNAs with G-starting anticodons in the fourfold degenerate codon boxes are sometimes absent [14], they are a part of the tRNA sets of most organisms in all three domains (although the G-starting anticodon often has turned into a I-starting anticodon in Eucarya). We conclude that the feature of having at least two tRNAs in each codon box (except the UAN codon box with two stop codons) was already present in LUCA. The degree of resemblance of the tRNA sets of the three domains is too high to make convergent evolution an acceptable alternative.

Another aspect that is found in all three domains is: **tRNAs with C-starting anticodons in addition to tRNAs with U-starting anticodons**. In the same way that the U-starting tRNA in a fourfold degenerate codon box is assisted by a tRNA with a G-starting anticodon, tRNAs with U-starting anticodons are assisted by a tRNA with a C-starting anticodon to obtain better reading of the G-ending codon. The feature of having both a C-starting anticodon and a U-starting anticodon working on the R-ending codons (except in the UAA codon box, the UGA codon box, and the AUA codon box, where stop and start signals complicate the situation) also is a universal property of living cells. Again, convergent evolution does not seem the most parsimonious hypothesis.

Third, we consider an aspect which is **not** found in all three domains: **tRNAs with I-starting anticodons**. The base modification *inosine* is found used in the CGN codon box of bacteria (see below). Use of I in the first anticodon position in other codon boxes of bacteria is very rare, but its use in the CGN codon box is standard in bacteria. In Eucarya, inosine in the first anticodon position is used in many codon boxes. Especially in the fourfold degenerate codon boxes it is the most frequently used nucleotide (see e.g. [28]). However, inosine is **not** a universal aspect of life. This base modification is absent from Archaea. We propose that the most parsimonious explanation is that LUCA did not have inosine, that Archaea never acquired inosine, that Bacteria evolved inosine, and that Eucarya inherited inosine from Bacteria.

Another aspect that is also **not** found in all three domains is: **tRNAs with xo**^**5**^**U-starting anticodons**. This modification, which enlarges rather than restricts the base pairing characteristics of U-starting anticodons, is exclusively bacterial. While some anticodon modifications are universal characteristics of living cells (e.g. the use of the Um modification enabling UUA recognition without suppression of UUY, or the use of the thio-modification enabling CAA, AAA, and GAA recognition without suppression of CAY, AAY, and GAY), other anticodon modification uses are domain-specific characteristics (e.g. abundant use of I-starting anticodons in Eucarya and use of xo^5^U-starting anticodons in Bacteria).

**Fig 3:**
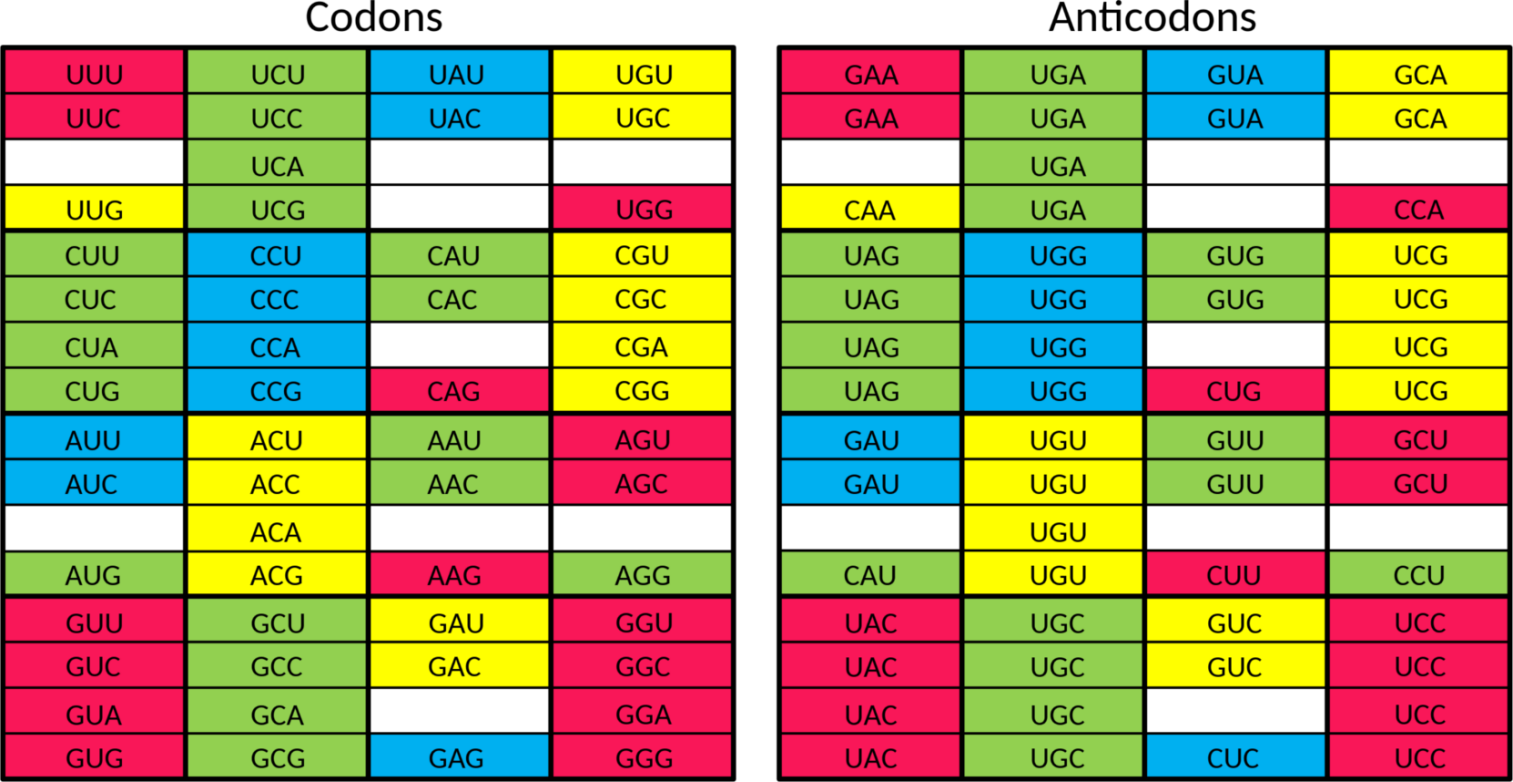
The proposed 23 tRNA stage in the evolution of the standard genetic code. Red, yellow, green, and blue are used to group codons together which are read by a single anticodon.

Taking into account the above four points, we propose that the set of 23 tRNAs lacking anticodon modifications (Fig 3) evolved into a stage in which the cell had a tRNA set containing **32 anticodons**. As described below, the set of 23 tRNAs [10] can be elegantly enlarged to this 32 tRNA set when the system has grown in sophistication, and the cellular machinery can support a substantially larger genome size.

**Fig 4:**
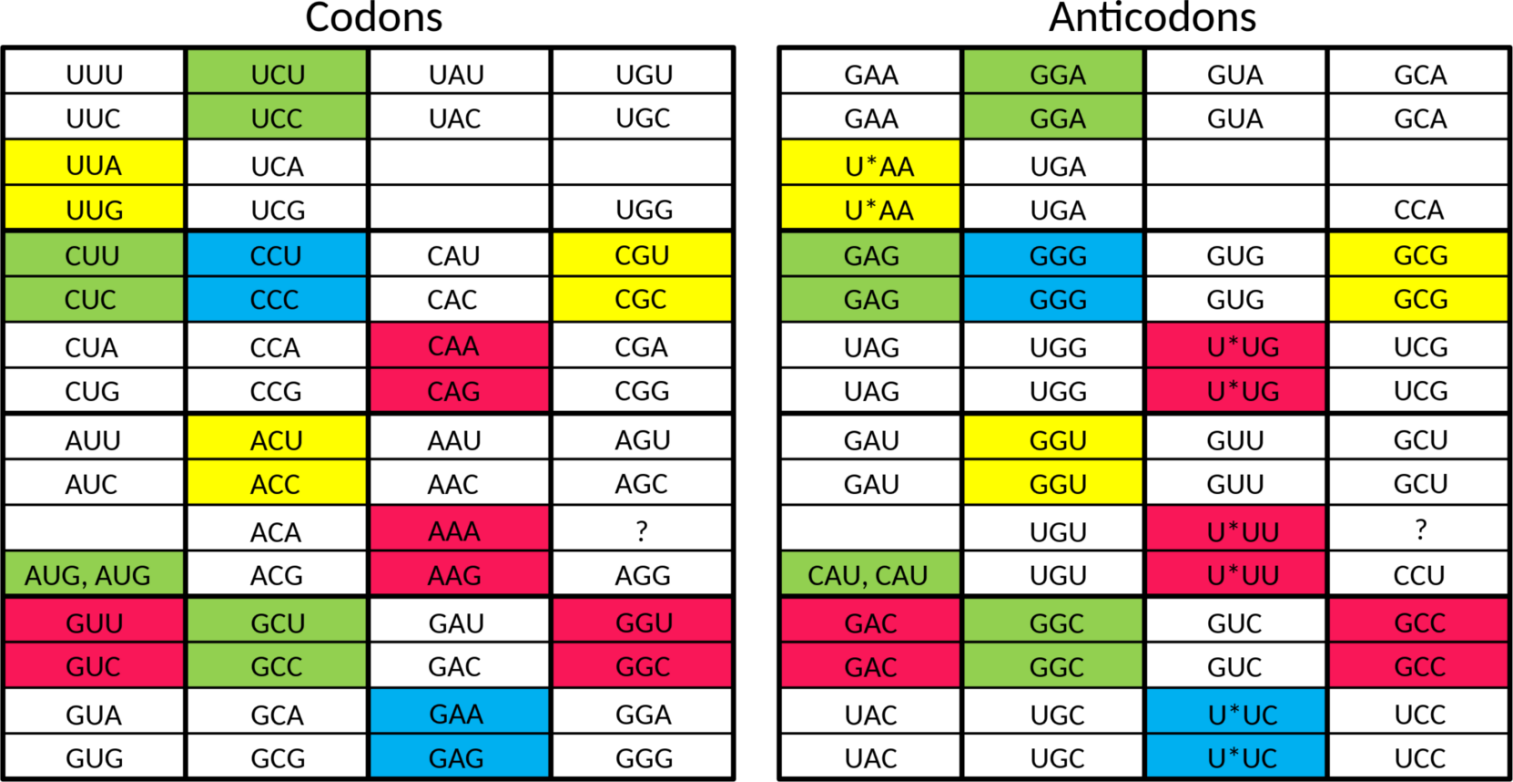
The proposed 32 tRNA stage in the evolution of the standard genetic code. The asterisks indicate anticodon first position modifications which are necessary to unambiguously read the respective codon box. At this stage there already is a distinction between initiator methionine and elongator methionine. Red, yellow, green, and blue are used to indicate the codons where changes happened compared to the situation in the previous figure.

This 32 tRNA set is essentially the tRNA set with G-starting anticodons for the Y-ending codons and U-starting anticodons for the R-ending codons. Because of initiation and termination of translation, three split codon boxes do not have a U-starting anticodon: UAN, UGN, and AUN (as stated above, we leave the question open if AGG was read by a CCU or a UCU anticodon in this stage). In the UAN codon box, the UAR codons are stop codons. Therefore, a single (G-starting) anticodon suffices to recognize the sense codons of the UAN codon box. In the UGN codon box, the UGA codon is a stop codon. Therefore, not a U-starting anticodon but a C-starting anticodon translates the G-ending codon in this codon box. Two anticodons are thus present in this codon box: a G-starting anticodon for the Y-ending codons and a C-starting anticodon for the UGG codon. In the AUN codon box, AUG has become the start codon. Three anticodons are therefore present in this codon box: a G-starting anticodon for the Y-ending codons, a C-starting anticodon for the AUG codon playing a role during translation initiation (see below), and a second C-starting anticodon for AUG codons specifying methionine during translation elongation. Please note that in the 32 tRNA stage of the genetic code no anticodon is able to read the AUA codon efficiently and unambiguously. In summary, the UAN, UGN, and AUN codon boxes have on average two anticodons per codon box, just as the remaining 13 codon boxes. Therefore, a set of 32 tRNAs (see Fig 4) is involved in more sophisticated translation (with anticodon modifications playing a role in the UUN, CAN, AAN, and GAN codon boxes) when compared to the 23 tRNA set discussed above. It is a tRNA set which has clearly progressed from the situation where limits of genomic memory space enforced superwobbling in fourfold degenerate codon boxes through a set of 23 tRNAs with unmodified anticodons. The first distinct step during stage 2 of the evolution of the SGC is the growth of the tRNA set from 23 tRNAs to 32 tRNAs.

**Fig 5:**
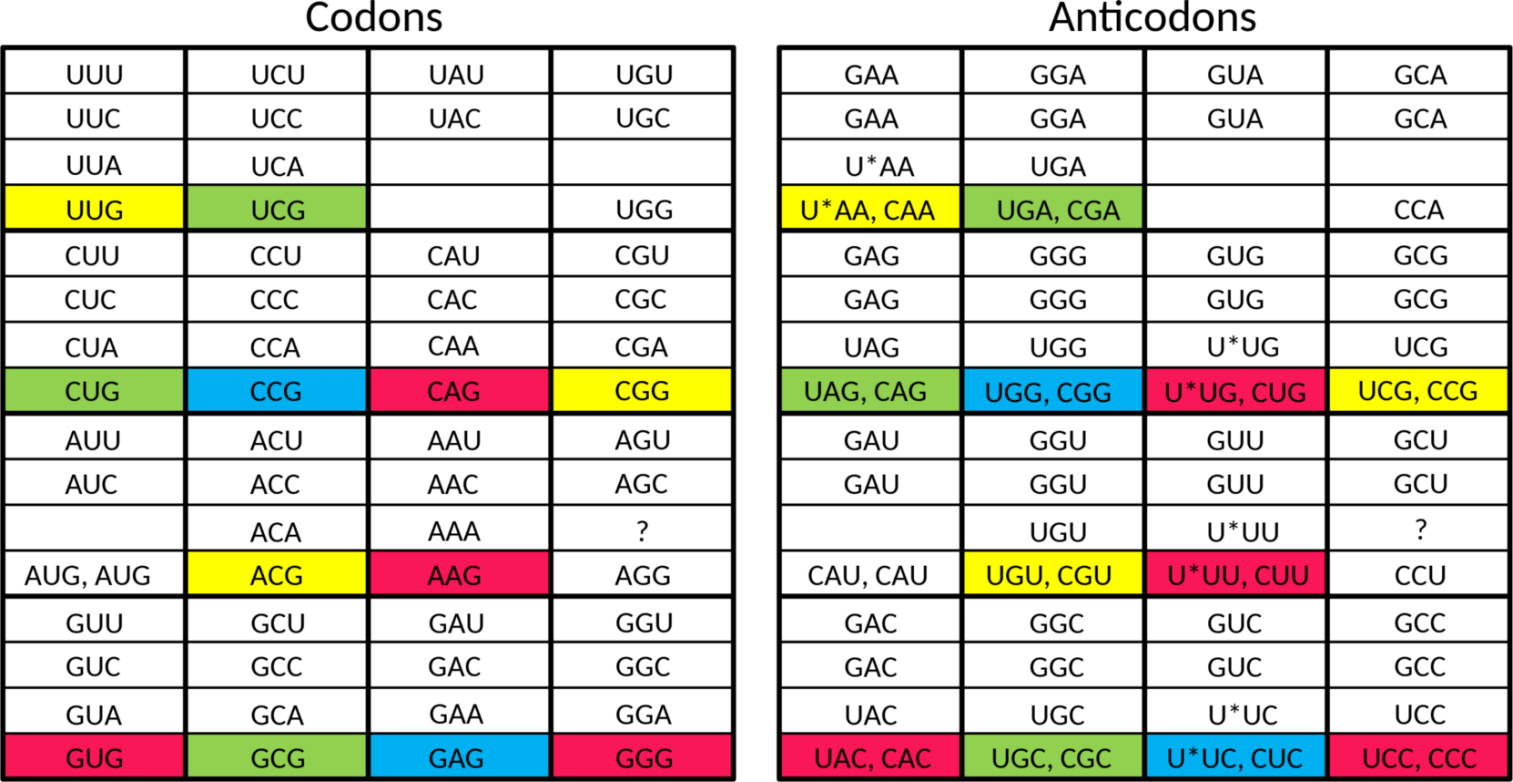
The proposed tRNA set of LUCA. The asterisks indicate anticodon first position modifications which are necessary to unambiguously read the respective codon box. At this stage there already is a distinction between initiator methionine and elongator methionine. Red, yellow, green, and blue are used to indicate the codons where changes happened compared to the situation in the previous figure.

As a next step, we propose a stage in which the cell had a tRNA set containing **44 or 45 anticodons**. We expect the following set of tRNAs to have been present in LUCA (Fig 5). For the eight fourfold degenerate codon boxes, we expect 3 tRNAs for each codon box (one with a G-starting anticodon, one with a U-starting anticodon, and one with a C-starting anticodon), which adds up to 24 tRNAs. For the five split codon boxes with 2 amino acids in the codon box, each encoded by two codons, we expect also 3 tRNAs for each codon box (one with a G-starting anticodon for the first amino acid, and for the second amino acid two tRNAs: one with a C-starting anticodon and one with an U-starting anticodon, the last one likely requiring anticodon modification to prevent misreading of the Y-ending codons). This adds up to 14 or 15 tRNAs (depending upon the open question if a tRNA with an UCU anticodon was present in this stage). For the two split codon boxes with 2 amino acids coded in the codon box, of which the second amino acid is encoded by just one G-ending codon, we expect 2 tRNAs (one with a G-starting anticodon for the first amino acid and one with a C-starting anticodon for the second amino acid). This adds up to 4 tRNAs. For the UAN codon box, we expect 1 tRNA (with a G-starting anticodon, the other two codons are recognized by a release factor protein). Finally, because the specialized initiator tRNA is universal (see e.g. [70]), we expect that one (with a C-starting anticodon) also. This makes a grand total of 24 + 14 or 15 + 4 +1 + 1 = 44 or 45. This second distinct step during stage 2 of the evolution of the SGC is the growth of the tRNA set from 32 tRNAs to 44 or 45 tRNAs.

**Fig 6:**
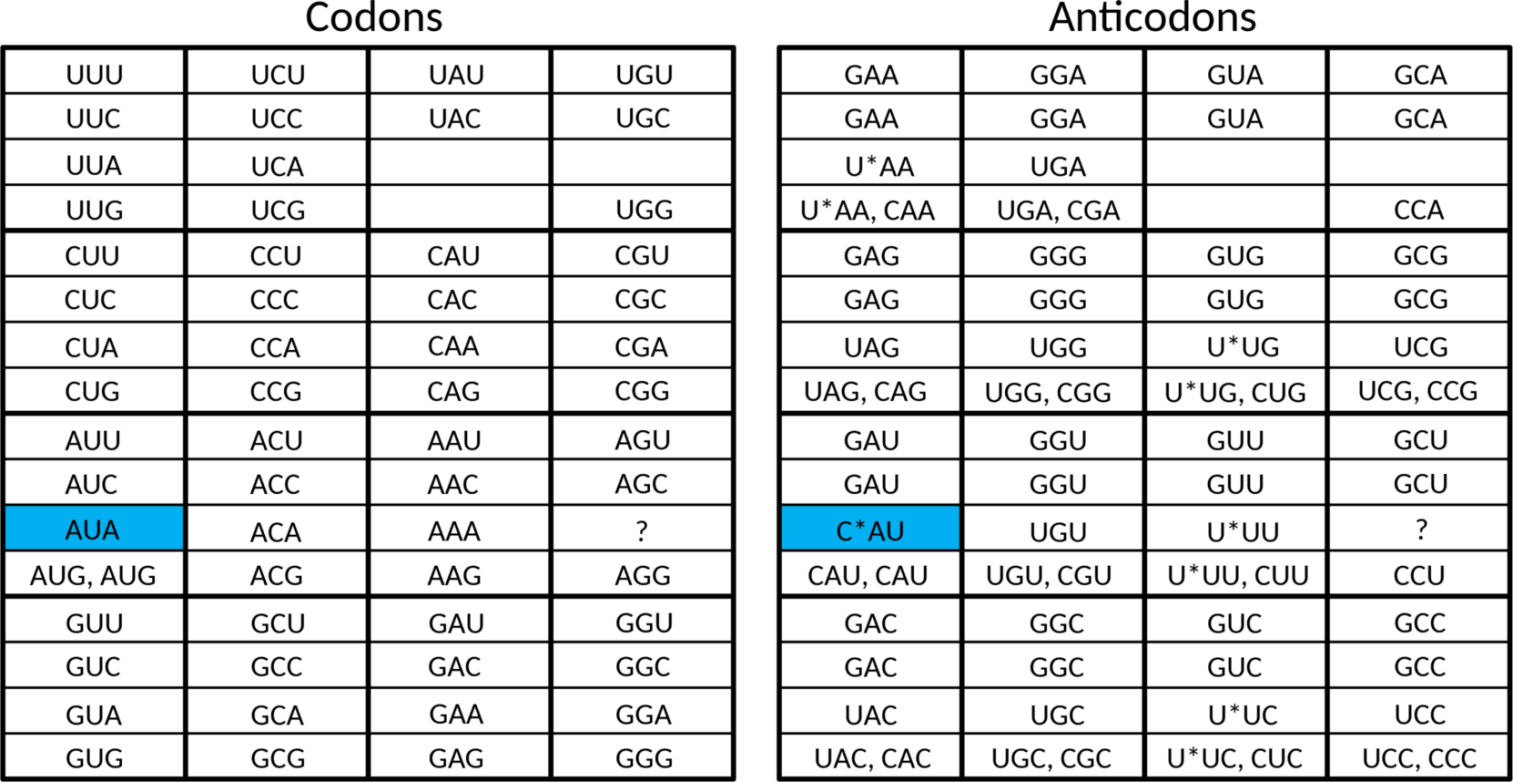
The tRNA set of present-day Archaea. The asterisks indicate anticodon first position modifications which are necessary to unambiguously read the respective codon box. The only difference with the proposed tRNA set of LUCA is the presence of the codon AUA in this codon repertoire (indicated by coloring with blue).

The difference between this proposed tRNA set of LUCA (Fig 5) and the one of present-day Archaea (Fig 6) is the presence of the second tRNA^Ile^ with an agmatidine-modified CAU anticodon in the latter. The third distinct step during stage 2 of the evolution of the SGC as mentioned above is the growth of the tRNA set to include a tRNA which can recognize the AUA codon. This third step is a post-LUCA development, and here we see convergent evolution in archaea and bacteria to obtain the capability of using AUA. This evolutionary challenge was solved in molecularly different ways (see [10, 44–47]).

**Fig 7:**
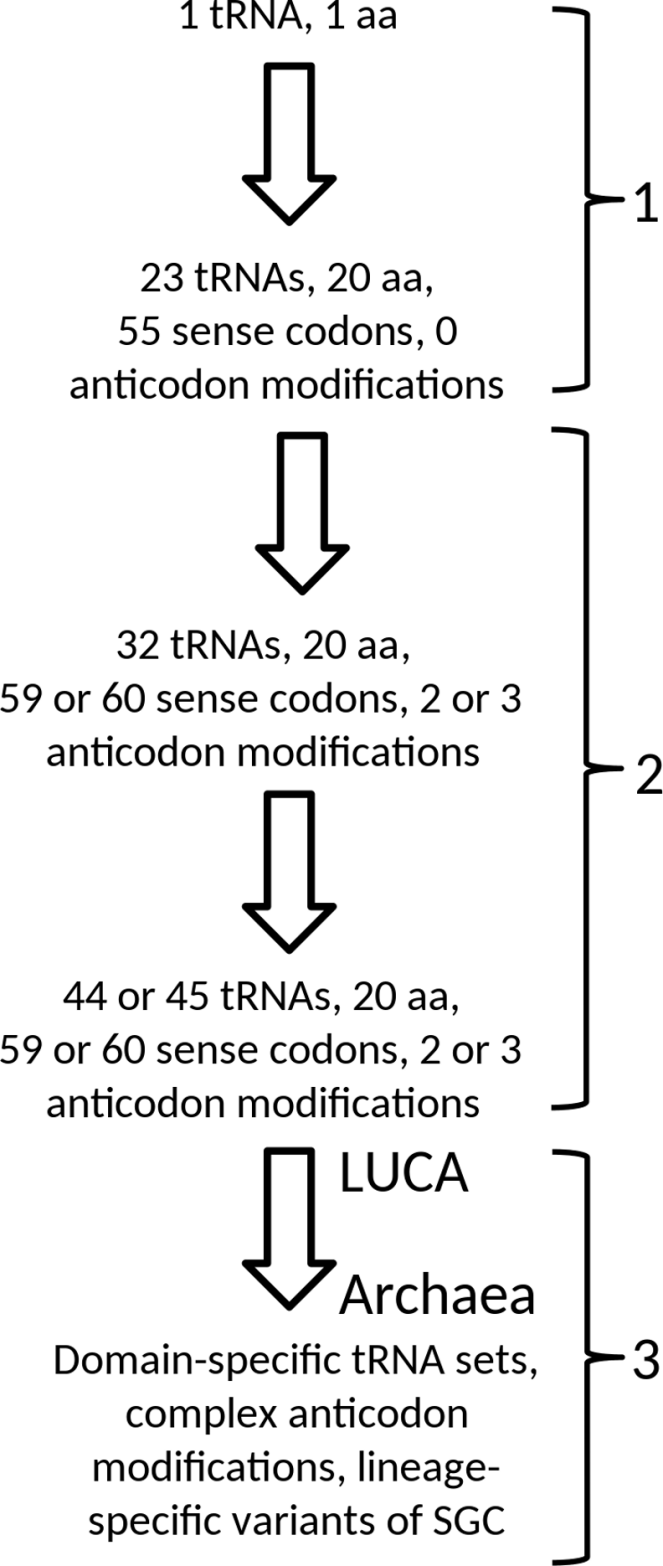
Summary of the proposed three stages in the evolutionary development of the tRNA set in the standard genetic code.

In Fig 7 we summarize the evolutionary development of the tRNA set, from a situation with one tRNA, via a situation with 23 tRNAs with unmodified anticodons, and subsequently a situation of 32 tRNAs, of which 4 or 5 carry an anticodon modification (2 or 3 different types of modification) to the 44 or 45 tRNA set in LUCA. We also indicate that unambiguous and efficient recognition of AUA was a “post-LUCA stage 2” development, which evolved convergently in Archaea and Bacteria. Please note that the introduction of unambiguous and efficient AGA recognition, presented as a “pre-LUCA stage 2 event” in our scheme, currently is an open question (see above). It is a possibility that LUCA was an organism which was significantly more complex than the organism with the 44 or 45 tRNAs, that the 44 or 45 tRNA stage was the one of a progenitor of LUCA, and that, after the more complex LUCA stage, streamlining the system did lead to a more simple tRNA set as found in Archaea. However, we consider the viewpoint of gradual growth in complexity the more parsimonious one.

### The tRNA sets of Bacteria, Archaea, and Eucarya differ from each other in a fundamental way [71]

Above we already emphasized the non-universal distribution of inosine and xo^5^U modifications. In summary, Eucarya often use inosine in the fourfold degenerate codon boxes. Inosine should be seen as a modification of A, because it emerges in nucleic acid strands as a result of enzymatic deamination of A. Archaea do not use inosine, while Bacteria only use it in three codon boxes: CGN and (only very rarely) CUN and ACN [27]. But, characteristic for Bacteria, a specific modification of U (xo^5^U_34_) is used for many tRNAs [71]. Importantly, these specific modification systems (A-deamination in Eucarya and a specific U-modification in Bacteria) leave a characteristic “fingerprint” in the codon usage of Eucarya and Bacteria [71]. Based on the comparatively restricted set of anticodon modification enzymes in Archaea and the resulting archaeal codon usage, we follow the authors of [71] in proposing that the situation in Archaea can be considered as the primordial situation (both in tRNA set and codon usage), from which Bacteria and Eucarya have diverged. While the A-deamination modification enzymes of Eucarya and the specific U-modification enzymes of Bacteria have strongly affected their codon usage, the codon usage of the Archaea has coevolved with a much more restricted modification pattern [71]. Woese’s three domains can thus be recognized in the codon usage of the three different kinds of cellular organisms, as demonstrated in [71]. Considering the primordial situation found in Archaea with respect to both tRNA set and codon usage, the primordial character implied by the name Archaea turns out to be very appropriate.

Elaborating on the notion that the tRNA set of present-day Archaea resembles the ancestral situation, Novoa et al. [71] refer to **Methanococcus-like Archaea** and describe that the tRNA set of this group of archaea is smaller than those of Non-Methanococcus-like Archaea. This observation led them to propose that the tRNA set in Methanococcus-like Archaea resembles the ancestral tRNA set. However, more sophisticated analysis (including the use of large data sets of concatenated sequences of informational proteins combined with the use of procedures to remove proteins that have been affected by lateral gene transfer) has indicated that these small-genome Archaea contain a *reduced* tRNA set derived from a relatively recent Archaeal ancestor (see e.g. [72,73]). This analysis establishes that the Non-Methanococcus-like tRNA set is the primordial one, while the (smaller) Methanococcus-like tRNA set is a derived one. Therefore, the 32 tRNA set presented above (which resembles the Methanococcus-like tRNA set) was the tRNA set of an *ancestor* of LUCA, just as the 23 tRNA set discussed earlier. Except for the G-ending codons in the UAN, UGN, and AUN codon boxes, all the G-ending codons (with the possible exception of AGG) in the primordial, Non-Methanococcus-like tRNA set stage were translated by a dedicated C-starting anticodon tRNA to *assist* a tRNA with an U-starting anticodon with the recognition of the G-ending codons (see e.g. [27]), because those codons were always less efficiently recognized by the U-starting anticodons (either modified or unmodified) than the A-ending codons. In some Archaeal lineages, a *reduction* in the number of tRNA genes occurred compared to the situation in LUCA. This process resembles the well-established case of the reduced tRNA set in mitochondria.

Nelson-Sathi and co-workers [74] recently reported that massive Bacteria-to-Archaea lateral gene transfer events are at the root of more than 10 major taxa within the Archaea. This finding implies that it is difficult to derive the genome of the “primordial archaeon”, and has the potential to complicate the proposal that the Archaeal tRNA set resembles the primordial tRNA set in LUCA. However, despite this massive gene transfer, the Archaea have retained their distinct character with respect to their tRNA set and codon usage. The laterally transferred bacterial genes, which allowed the archaeon in which they were incorporated to conquer a new ecological niche, subsequently adjusted their codon usage to the archaeal system. This consideration indicates that the tRNA set is one of the most stable characteristics of a cell. Novoa and co-workers [71] already reached this conclusion with respect to individual tRNA genes: the sequences can undergo lateral gene transfer, but the *functions* (having a tRNA with a specific anticodon delivering a specific amino acid) needed to be continuously fulfilled. This stability of function is also relevant for the U-thiolation (see above) necessary for unambiguous codon reading in the CAN, AAN, and GAN codon boxes: not *the* gene for the enzyme is continuously present, but *a* gene for *a* U-thiolation enzyme. Vetsigian and co-workers [75] already placed emphasis on the need during the development of the genetic code for an “innovation-sharing protocol” to be able to incorporate foreign DNA to re-gain functions lost due to mutation, and to gain new functions needed for survival in an innovation-developing competitive environment. Considering the results of Nelson-Sathi et al., [74], the Archaea appear to have retained this genomic flexibility to a greater extent than the Bacteria and the Eucarya.

Previous published work [76] is relevant to the resemblance of the tRNA set of archaea to that of the LUCA discussed here. In a study of tRNA paralogs, Xue and co-workers showed that archaeal tRNAs are less divergent than others. However, the conclusions drawn from this observation have attracted substantial debate, see e.g. [77–79]. For the arguments against *Methanopyrus kandleri* being an organism which is primitive compared to other archaea, see [72]. For the arguments that small genome and small tRNA set archaea are generally organisms with a reduced genome rather than primitive organisms, see [73]. While one has to be extremely careful in interpreting evolution concerning archaea with reduced genomes, the fact that archaea in general have slowly evolving tRNAs is emerging as an important conclusion.

Different views on evolution of the tRNA sets in the three domains have been proposed. One view is that the set of 20 amino acids evolved independently in the three domains in a convergent manner [27]. The diversity of the tRNA anticodon modifications found among living cells has been brought forward as support for this view. Another view is that LUCA already functioned with the canonical set of 20 amino acids (as is argued here). The latter view is supported by the fact that no modifications are necessary to unambiguously read 55 of the 61 sense codons while encoding all 20 canonical amino acids. Based on the work of Lehmann and Libchaber [11], the conclusion can be drawn that 8 tRNAs with unmodified U-starting anticodons suffice to read 32 of the sense codons. To have unambiguous coding in the remaining codon boxes, the exclusive use of tRNAs with unmodified G-starting anticodons and unmodified C-starting anticodons (simply behaving according to the wobble rules as proposed by Crick [21]) suffices. The actual amino acid assignments in the standard genetic code allow such a mechanism to provide relatively efficient and relatively unambiguous encoding of all 20 amino acids.

The fact that the comparatively simple tRNA set of the Archaea is closer to this proposed ancestral situation than the comparatively more derived and complex tRNA sets (especially concerning their anticodon modification patterns) of Bacteria and Eucarya supports this view. Based on the fundamental behavior of unmodified anticodon function as presented in [21] and [11], we thus conclude that the tRNA set of the Archaea is closer to the primordial tRNA set. In addition, we argue that the modification enzyme thiouridylase predates LUCA (providing translation of 3 of the missing codons: CAA, AAA, and GAA, without introducing ambiguity by misreading of Y-ending codons). We expect that LUCA also already used the methylation of the ribose part of U_34_ of anticodon UAA, which enabled the use of UUA. As presented in recent literature [10,46,47], accurate AUA decoding is a convergent development in Archaea and Bacteria. We leave the usage of the AGA codon in LUCA as an open question. We conclude that except for AUA (and possibly AGA) LUCA already used all sense codons, and was able to do so using only two (or possibly three) relatively simple anticodon modifications.

The analysis presented here reveals that the second stage of genetic code development (acquiring the ability to recognize all 61 sense codons quickly and unambiguously) was nearly complete in LUCA. Only one or two sense codons were outside LUCA’s sense codon repertoire. In summary, this paper contains two main messages. Firstly, the importance of the extremely regular structure of the genetic code for understanding the evolution of life is brought into focus. Secondly, LUCA greatly resembled present-day Archaea in terms of its tRNA set, while Bacteria and Eucarya have diverged from this situation.
